# The Impact of Virtual Reality as a Rehabilitation Method Using TRAVEE System on Functional Outcomes and Disability in Stroke Patients: A Pilot Study

**DOI:** 10.3390/biomedicines12112450

**Published:** 2024-10-25

**Authors:** Claudia-Gabriela Potcovaru, Delia Cinteză, Miruna Ioana Săndulescu, Daniela Poenaru, Ovidiu Chiriac, Cristian Lambru, Alin Moldoveanu, Ana Magdalena Anghel, Mihai Berteanu

**Affiliations:** 1 Physical Medicine and Rehabilitation, “Carol Davila” University of Medicine and Pharmacy, 050474 Bucharest, Romania; claudia-gabriela.potcovaru@drd.umfcd.ro (C.-G.P.); daniela.poenaru@umfcd.ro (D.P.);; 2 National Institute of Rehabilitation, Physical Medicine and Balneoclimatology, 030079 Bucharest, Romania; 3 Computers Department, Faculty of Automatic Control and Computers, National University of Science and Technology Politehnica Bucharest, 060042 Bucharest, Romania; andrei.lambru@upb.ro (C.L.); alin.moldoveanu@cs.pub.ro (A.M.); 4 Automatic Control and Applied Informatics Department, Faculty of Automatic Control and Computers, National University of Science and Technology Politehnica Bucharest, 060042 Bucharest, Romania

**Keywords:** stroke, rehabilitation, disability, WHODAS 2.0, upper limb, virtual reality

## Abstract

Background: Stroke is the third leading cause of disability. Virtual reality (VR) has shown promising results in post-stroke rehabilitation. The VR TRAVEE system was designed for the neuromotor rehabilitation of the upper limb after a stroke and offers the ability to track limb movements by providing auditory feedback and visual augmentation. The World Health Organization Disability Assessment Schedule 2.0 (WHODAS 2.0), aligned with the International Classification of Functioning, Disability, and Health (ICF) principles, is a valid tool for measuring disability regardless of its cause. This study aimed to investigate the feasibility of the VR TRAVEE system in upper limb rehabilitation for stroke patients. Methods: A total of 14 stroke patients with residual hemiparesis were enrolled in the study. They underwent a 10-day program combining conventional therapy (CnvT) with VR rehabilitation. At baseline (T0), the upper limb was assessed using the Modified Ashworth Scale (MAS), active range of motion (AROM), and the Numeric Rating Scale (NRS) for pain. These assessments were repeated after the 10-day rehabilitation program (T1). Additionally, disability was measured using WHODAS 2.0 at T0 and again 30 days after completing the program. Results: Significant improvements were observed in AROM and MAS scores for the shoulder, elbow, wrist, and metacarpophalangeal joints, as well as in the reduction in shoulder pain (*p* ˂ 0.001). WHODAS scores decreased across all six domains, with a statistically significant improvement in the Cognition domain (*p* = 0.011). Conclusions: Combining CnvT with VR as a rehabilitation approach enhances motor function in the upper limb. This method has the potential to reduce disability scores and promote neuroplasticity.

## 1. Introduction

The second cause of death worldwide and the third leading cause of Disability-Adjusted Life Years (DALYs) is represented by stroke, a common health issue that often leads to disability [1]. Recently, stroke has been associated with a substantially incremental burden that increased from 1990 to 2019, with low- and middle-income countries contributing most significantly with an increase of 70% in incidence, 102% in prevalence, 43% in death rate, and, respectively, 143% increase in DALYs [2]. Worldwide, the incidence of stroke is increasing in young and middle-aged people (<55 years) [3]. Even though most patients survive a stroke, the long-term deficits caused by motor, sensory, and cognitive impairments lead to diminished functionality, activity limitation, and participation restrictions with significant impact on patients and their families [4,5].

To help the patient regain a level of functioning as close as possible to that that was prior to the event, a large portion of the medical rehabilitation team’s effort is directed toward motor rehabilitation. Changes in motor ability can be achieved through three different pathways: restitution, substitution, or compensation. This is very important because motor impairment is the main effect of stroke and affects about 80% of stroke survivors [6]. This is due to a lack of central nervous system control over the muscles and movements in one side of the body’s face, hand, and leg muscles, which limits mobility and affects the patient’s functional abilities [7,8].

Levin et al. differentiated between motor compensation and motor rehabilitation based on the International Classification of Functioning, Disability, and Health (ICF). They concluded that motor rehabilitation included the successful completion of tasks performed, the restoration of lost function in neural tissue, and the ability to perform movement, all in the same way as prior to the event. In these areas, motor compensation can take several forms, such as the brain tissue learning a new function that was absent before the damage, performing the movement in a novel fashion, and utilizing alternative methods to complete tasks [9]. Following a brain injury, the neurons in the affected areas must be reorganized to facilitate the recovery of motor functions. Behavior is a result of this restructured brain activity, and the process of learning new skills promotes adaptive neural restructuring, which in turn enhances brain plasticity. Repetition alone, however, does not determine reorganization. This occurs only when learning is a part of the experience. Thus, the goal of motor rehabilitation should be to promote plasticity in patients by giving them experiences that test their motor abilities. Furthermore, it has been demonstrated that motor learning concepts like intensity, repetition, task orientation, and feedback influence how well a person functions following a stroke [10,11,12].

Virtual reality (VR) enables the construction of computer-generated environments and provides individualized experiences with respect to three senses: touch, sound, and vision [13]. Studies on the use of VR for post-stroke motor therapy show encouraging outcomes, especially for upper limb rehabilitation. Reaching, gripping, carrying, and releasing things in a virtual environment all follow similar kinematics to real-world movements, which suggests that practicing arm movements in VR can be a practical substitute [14]. VR has also been successfully used to improve distal motor control but is usually combined with conventional therapy (CnvT) or robotic-assisted therapy [15,16]. It has been proposed that proprioceptive feedback can take advantage of the multimodal characteristics of goal-oriented movement monitoring and self-awareness feedback. Goal-oriented proprioceptive training can influence balance and autonomy, and VR can facilitate the use of walking aids, thereby positively influencing balance [17]. However, without particularly addressing hand and finger dexterity, clinical research on these systems has, up to this point, mostly focused on shoulder and elbow training [18].

The TRAVEE system has been in existence since 2019 and has recently undergone a substantial update. The TRAVEE system offers patients the opportunity to perform upper limb exercises through gaming using VR. The system utilizes a Meta Quest 2 VR headset, a commercial product developed by Reality Labs, a research unit with locations worldwide and a subsidiary division of Meta Platforms, Inc., headquartered in Menlo Park, CA, USA, which provides the capability to automatically track limb movements from the wrist to the hands and fingers. The system also offers visual and auditory adjustments to diversify the virtual world context in which the patient performs game-based exercises. In addition to the set of game-based exercises that can be performed in the virtual world, the TRAVEE system offers therapists a control panel where they can configure specific properties for each exercise. This panel has a web-based graphical interface that is accessed via a web client running on a laptop. Additionally, to view the content the patient sees in the virtual world, a casting window displaying the VR headset’s image is launched alongside the control panel interface [19].

Following a stroke, rehabilitation can be challenging and often unclear. Numerous randomized clinical trials combine diverse rehabilitation techniques, such as CnvT, robot-assisted arm training, and VR therapy, based on the principles mentioned above. Motor rehabilitation is difficult to assess; the range of motion (ROM) can be improved, but this might not result in any overall functional gains [20]. Individualized rehabilitation programs that address all deficits remaining after a stroke, with a particular focus on motor deficits, should be designed in a SMART (Specific, Measurable, Achievable, Relevant, and Time-bound) manner based on Goal Attainment Scaling (GAS) [21]. Additionally, assessing health and disability remains challenging. To address this, the World Health Organization (WHO) developed the WHO Disability Assessment Schedule 2.0 (WHODAS 2.0) to evaluate disability and health independent of the underlying medical cause [22,23].

The aim of this study is to test a VR system designed for stroke survivors’ rehabilitation that is based on the principles of motor learning and neural plasticity, with the goal of promoting neuromotor rehabilitation. The benefits of VR therapy were measured using the WHODAS 2.0 and ROM. A secondary goal was to evaluate the acceptance of this intervention among both patients and therapists.

## 2. Materials and Methods

### 2.1. Study Design, Protocol, and Patients

The present study was conducted in accordance with the principles of the Declaration of Helsinki and received approval from the Institutional Ethics Committee of the Romanian National Institute of Rehabilitation, Physical Medicine, and Balneoclimatology, with the registration number code NRCRT6/18.09.2023 and the date of approval of 18 September 2023. All patients completed a written informed consent.

This is a single-center cohort study involving patients admitted for a stroke rehabilitation program between 5 October 2023 and 1 July 2024 who completed a 10-day CnvT combined with a VR rehabilitation program. To be included in this study patients had to meet the following inclusion criteria: (1) patients with chronic ischemic or hemorrhagic stroke (confirmed by a CT/MRI brain scan); (2) moderate to severe deficit of the paretic upper limb (measured by active ROM); (3) no cognitive deficits as assessed by the Mini-Mental State Examination (MMSE ≥ 25); (4) cooperative behavior; (5) age ˂ 80 years. The exclusion criteria were as follows: (1) other neurological condition that results in paresis of the upper limb; (2) cognitive deficits (MMSE ≤ 25); (3) inability to understand instructions; (4) other psychiatric impairments that limit the ability to participate or give informed consent; (5) patients who did not respond to WHODAS 2.0 questionnaire reevaluation after 30 days.

From the patients’ medical histories, data regarding the type of stroke (ischemic or hemorrhagic), the time passed since the onset of stroke, comorbidities (the presence or absence of high blood pressure (HBP), type 2 diabetes mellitus (T2DM), dyslipidemia, and atrial fibrillation (AF)) were collected. Dyslipidemia was considered if they were undergoing treatment with lipid-lowering therapy or if they had a total cholesterol level over 200 mg/dL or low-density lipoprotein cholesterol (LDL-C) greater than 130 mg/dL. HBP was defined as having systolic blood pressure (SBP) or diastolic blood pressure (DBP) ≥ 140/90 mmHg. Patients were considered to have T2DM if they were receiving hypoglycemic medication or if their glycated hemoglobin (HbA1c) was ≥6.5%. Patients were considered with AF if they had an episode on electrocardiogram of AF. Additionally, information on the patients’ alcohol and smoking habits (whether they were smokers or non-smokers) and their dominant hand was collected through direct questioning. Alcohol consumption was measured using an online calculator recommended on the internet [24]. Less than 7 units of alcohol per week for women and 14 units per week for men were considered a normal dosage and were categorized as non-alcohol usage [25].

The patients were evaluated at two different times: at admission in the rehabilitation unit (T0), before the rehabilitation program was applied, and after completing 10 days of CnvT combined with VR rehabilitation (T1), and again for disability assessment with WHODAS 2.0 questionnaire at 30 days after finishing the 10 days rehabilitation program.

At T0, patients were assessed using the Modified Ashworth Scale (MAS) for shoulder abductors, elbow flexors, pronators, and finger flexors. Additionally, active range of motion (ROM) was evaluated for shoulder abductors, shoulder flexors, internal and external shoulder rotation, elbow flexors, pronation, supination, wrist extension and flexion, and flexion and extension of the metacarpophalangeal joint. Shoulder pain was measured using the Numeric Rating Scale (NRS). The WHODAS 2.0 questionnaire was also administered to assess disability and functioning.

At T1, after completing the 10-day rehabilitation program, these assessments were repeated, including MAS, ROM evaluations, and NRS for shoulder pain.

At 30 days post-discharge, patients were contacted via telephone and reassessed with the WHODAS 2.0 questionnaire to evaluate their long-term progress.

### 2.2. CnvT and VR Rehabilitation Program

Specific to each rehabilitation department center, CnvT represents a variety of therapies and rehabilitation procedures. For the patients, it included strength exercises, aerobic exercises, both passive and active range of motion (ROM) exercises, stretching, balance training, occupational therapy sessions, and speech therapy when needed. In addition to physical exercises, other physical agents were used, such as electrotherapy with electrical stimulation, pain management electrotherapy, ultrasound therapy, massage, and the application of splints or orthoses for spastic limb posture. The conventional therapy consisted of 1.5 h per day, divided into two sessions, 5 days/week, over a 10-day rehabilitation program. All patients followed the same program.

**Figure 1:**
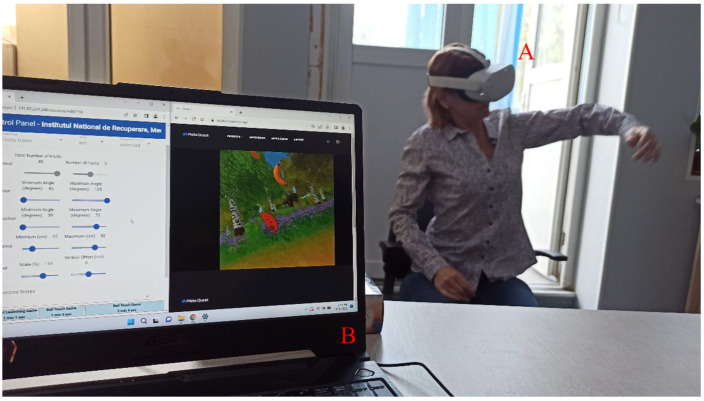
The use of the TRAVEE system by a patient, marked by the letter A, and by the therapist through the control panel running on the laptop marked with the letter B. In addition to the control panel interface, the therapist has access to the content viewed by the patient via a casting interface, visible on the laptop monitor in the image. The control panel allows the therapist to modify the parameters of the game-based exercise.

VR rehabilitation was carried out using the TRAVEE system, which is designed for neuromotor rehabilitation of the upper limb after stroke. The TRAVEE system incorporates multiple advanced technologies (Functional Electrical Stimulation, electromyography, visual and auditory feedback) that cover the rehabilitation needs of most patients. However, in this study, we utilized the Meta Quest 2 VR headset, (Meta Platforms, Inc., Menlo Park, CA, USA) which provides the capability to automatically track limb movements from the wrist to the hands and fingers. This way, patients can see their hands in the virtual world and synchronize their hand movements with specific tasks they need to accomplish without the need to hold a hardware device like a controller. During the exercises, the TRAVEE system provided auditory feedback and visual augmentation of the patient’s movements to enhance motivation during rehabilitation sessions [19,26]. In Figure 1, on the right, a patient using the TRAVEE system can be seen. The patient is wearing a Meta Quest 2 VR device (Meta Platforms, Inc., Menlo Park, CA, USA) and is performing a hand movement to complete a task in the virtual world.

There were 10 games that patients could play: Each game was designed to train different impairments of the upper limb and, at the same time, offer an engaging, immersive, and motivating experience. The games are summarized in the following Table 1.

Each game was designed to train different impairments of the upper limb. For instance, if the goal was to train more abduction and flexion of the shoulder, the selected games were the Fire game and the Rocket game. To train internal and external rotation of the shoulder, patients were assigned to the cooking game. For training the flexors and extensors of the elbow, patients were assigned to the Fruit games—specifically, the Fruit to Basket game for extension and supination and the Fruit to Mouth game for flexion and pronation. If the aim was to train supination and pronation, the patient played the Road game. Lastly, for training flexion and extension of the wrist, patients engaged in the Piano game and Diorama game.

### 2.3. Muscle Tone Assessment

The muscle tone of the upper extremity in the shoulder, elbow, and wrist was assessed using MAS. MAS is a numerical scale that grades spasticity on a scale of 0 to 4. A score of 0 indicates no increase in muscle tone, while a score of 4 indicates rigid flexion or extension. The scale includes an additional grade, 1+, which represents a slight increase in muscle tone, manifesting as a catch, followed by minimal resistance throughout less than half of the range ROM [27]. To ensure accuracy and reliability, two doctors trained in MAS evaluation independently assessed each patient. If there were any disagreements, they reached a consensus on the final score.

### 2.4. Range of Motion Joint Assessment

Range of motion (ROM) was assessed in the shoulder, elbow, wrist, and metacarpophalangeal joint. ROM was evaluated actively (AROM). ROM is the range of motion, expressed in degrees, that can be obtained at one or more joints and covers the start and finish of a movement in a certain plane. It shows the angle at which the joint travels from its anatomical location to its maximum range of motion. ROM was measured using a goniometer for each joint and delivered in degrees. AROM represents the arc of motion produced by the individual’s voluntary, unassisted muscle contraction [28].

### 2.5. Health and Disability Evaluation

The health and disability status of all patients was assessed using the WHODAS 2.0 questionnaire, published by WHO in 2010 which was administered through interviews conducted at admission and 30 days following the rehabilitation program. The 36-item version of WHODAS 2.0 was administered to patients who continued to work or engaged in school activities. The 32-item version was used for those who were retired or unemployed. The scoring of WHODAS 2.0 was performed using the complex scoring method available from the WHO website.

### 2.6. Statistical Analysis

The data were organized using Excel Office 365, Version 16.0, for Windows 10 and Gnu PSPP 1.4.1 software, which facilitated a thorough statistical assessment of the parameters. Patient characteristics were described as mean and standard deviation (SD) for normally distributed data or median and interquartile range (IQR) for non-normally distributed data. Categorical variables were described using frequencies and percentages. The distribution of the data was assessed using the Kolmogorov–Smirnov and Shapiro–Wilk tests. Variables with *p*-values greater than 0.05 in both tests were considered normally distributed, allowing for parametric analysis. For variables with *p*-values less than 0.05, non-parametric methods were applied. For normally distributed variables, paired sample *t*-tests were conducted to evaluate the significance of changes between baseline (T0) and post-intervention (T1) measurements. For non-normally distributed variables, the Wilcoxon Signed-Rank Test was used to assess changes between T0 and T1. Correlation analyses were performed using the Pearson correlation coefficient for normally distributed data and the Spearman correlation coefficient for non-normally distributed data. The scoring of WHODAS 2.0 was performed using the complex scoring method available from the WHO website [29]. *p* < 0.05 was considered statistically significant.

## 3. Results

**Figure 2:**
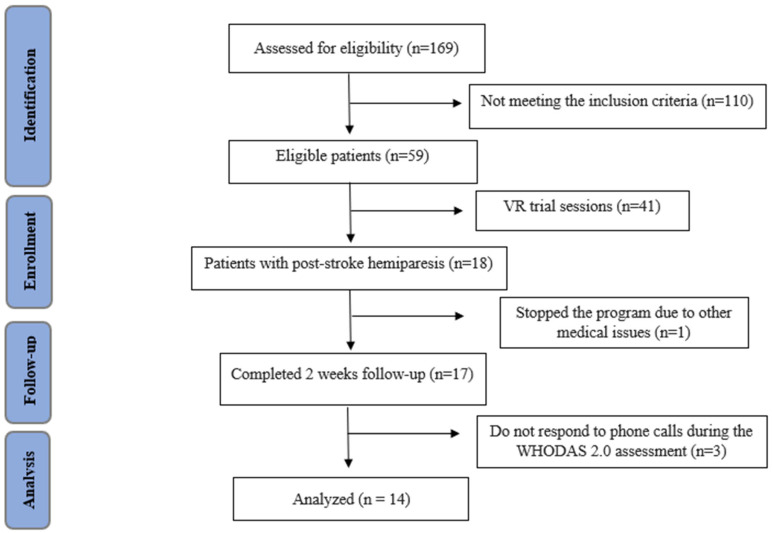
STROBE flow chart of the study. STROBE: STrengthening the Reporting of OBservational studies in Epidemiology.

A total of 169 consecutive inpatients were screened for eligibility criteria. Among them, 59 met the inclusion criteria and were deemed eligible. Of these, 41 patients participated only in trial sessions using the TRAVEE VR system. Subsequently, 18 patients with post-stroke hemiparesis were enrolled to complete a 2-week CnvT combined with a VR rehabilitation program. Of these, one patient discontinued the program due to a Clostridium difficile infection, which required transfer to a gastroenterology unit, and three patients did not respond to phone calls for the WHODAS 2.0 assessment at 30 days. Ultimately, 14 patients were included in the study and analyzed, as shown in Figure 2. The study included 12 males and 2 females, with a mean age of 55.29 ± 8.6 years. The participants had an average education duration of 13.36 ± 2.72 years. Regarding employment, 71.42% were retirees, while 28.57% continued with paid work. Most participants (78.57%, 11 patients) were from urban areas, and 21.42% (3 patients) were from rural areas.

In terms of lifestyle, 50% of the participants were smokers, and three patients (21.42%) consumed alcohol. All patients were right-handed. Stroke characteristics showed that 11 patients (78.57%) had ischemic strokes, and 3 patients (21.42%) had hemorrhagic strokes. Among those with ischemic strokes, two patients received thrombolysis treatment.

Most patients (85.71%, 12 patients) had left-sided involvement, while 14.29% (2 patients) had right-sided involvement. The median time since stroke onset was 26.25 months, with an interquartile range of 5.25 to 31.5 months. The average MMSE score was 27.57 ± 2.03. Additionally, eight patients received botulinum toxin injections, with a mean time of 1.81 months since the last injection, as seen in Table 2.

Regarding comorbidities, 13 patients had HBP (92.86%), 3 patients had T2DM (21.43%), 12 patients had dyslipidemia (85.71%), and 2 patients had AF (14.29%), as seen in Table 3.

The analysis of the Modified Ashworth Scale (MAS) for various muscle groups between T0 and T1 showed statistically significant reductions in spasticity for most of the measured endpoints, as seen in Table 4.

The analysis revealed statistically significant improvements in active range of motion (AROM) and reductions in Numeric Rating Scale (NRS) shoulder pain between T0 and T1, as seen in Table 5.

WHODAS 2.0 scores across all six domains are presented in Table 6, both at T0 (before the rehabilitation program) and 30 days after the completion of the combined individual rehabilitation program.

## 4. Discussion

Using immersive VR and, respectively, the TRAVEE system in association with CnvT as a rehabilitation technique on the upper limb motor impairment and functional outcomes in stroke patients with residual hemiparesis can lead to significant improvements in aROM, spasticity, and overall disability scores. Upper limb motor functions were evaluated using the MAS and AROM before and after 10 days of the combined rehabilitation program. Functional outcomes were assessed using the WHODAS 2.0 questionnaire administered before treatment and 30 days after completion. Existing data demonstrate that the combination of VR with CnvT improves both motor and cognitive functions, data that are similar to our results, where the Cognition domain (D1) decreases significantly after treatment [30]. Furthermore, the effectiveness of immersive VR is supported by its significant advantages in upper limb rehabilitation, as evidenced by improvements in motor impairment measured using the Fugl–Meyer Arm Scale and functional abilities assessed with the Box and Blocks Test and the Manual Function Test when compared to no therapy [31]. Moreover, studies show that, after a four-week follow-up, there is a significant difference in WHODAS 2.0 scores in stroke patients who underwent 270 min of CnvT combined with VR as a rehabilitation program [32]. This highlights the potential of VR to significantly improve motor functioning and overall recovery in stroke patients.

Being middle-aged, the patients did not have conditions commonly associated with older adults, such as hearing loss, cataracts, or other visual acuity issues like macular degeneration, which could otherwise be significant barriers to using new technologies. Additionally, it is likely that these patients had previous contact with new technologies and are more familiar with using them, rather than the older population, who may be less technologically adept [33]. The average education level suggests a higher capacity for understanding more complex instructions. The fact that most of the patients were married indicates they had support, were not living alone, and could receive help when needed. Most patients had the left side of their body affected by the stroke, but all were right-handed, indicating that the dominant hemisphere was not affected in most cases. Although 14.27% of patients had strokes affecting the dominant hemisphere, they did not exhibit significant aphasia, even though most studies indicate that strokes in the dominant hemisphere are often accompanied by aphasia [34]. The cohort studies have diverse comorbidities, such as HBP, T2DM, dyslipidemia, and atrial fibrillation, which can influence the rehabilitation program [35]. For instance, oxidative stress, which is commonly associated with these conditions, may impact the efficacy of rehabilitation. Physical activities and various supplements typically used in comprehensive rehabilitation programs can contribute to the reduction in plasma fasting glucose, serum oxidized low-density lipoprotein (LDL), and oxidative stress. This suggests that a rehabilitation program incorporating physical activity may help lower fasting glucose levels, reduce dyslipidemia, and decrease hypertension [36,37,38].

In our study, the MAS scores decreased significantly after 10 days of combining VR with CnvT in shoulder adductors, elbow flexors, pronators, wrist flexors, and finger flexors. These muscles were assessed because they are the most commonly affected by spasticity following a stroke, often corresponding to postural patterns type III and IV of spasticity as classified by Hefter [39]. Patients without spasticity have much better motor recovery and functional outcomes than patients with spasticity after rehabilitation programs, particularly in self-care, mobility, and sphincter control [40]. The median time since stroke onset is 26.25 (5.25 to 31.5) months, and studies show that spasticity increases within the first 12 months after the onset of stroke. Studies have reported a prevalence of 17% for spasticity one year after a first-ever stroke, with only 4% experiencing disabling spasticity [41]. Other studies have reported a prevalence of 42.4% of spasticity in patients admitted for stroke rehabilitation [40]. A total of 8 out of 14 patients were injected with botulinum toxin for spasticity, with the median time since the last injection being 1.81 months. The injections were administered only to spastic muscles if the spasticity affected the patient’s functionality, indicating that spasticity impacted the functionality of more than half of the patients. As a result, the reduction in spasticity demonstrated in our study could contribute to improvements in AROM and reductions in WHODAS 2.0 scores, thereby being associated with a decrease in disability. The VR TRAVEE system, when paired with occupational therapy, has the potential to improve upper limb function. However, more research is needed to evaluate whether VR can completely replace traditional occupational therapy. Despite technological developments, the presence of a therapist is still required, particularly for patients with high spasticity and poor motor control, to guide and support them in achieving complete ROM during exercises [42].

Our study focused on measuring AROM, as we are particularly interested in assessing what patients can achieve independently. Given that the median time since stroke onset is 26.25 (5.25 to 31.5), we first aim to improve the functionality of our patients. Study shows that in the acute phase (the first three months), patients who received PROM exercises showed significant improvements in motor function, as measured by muscle strength using the Oxford Scale, compared to those who received CnvT without specific PROM [43]. For recovery after stroke, it is better to use both PROM and AROM exercises. AROM exercises are more effective in improving motor function and muscle strength when patients can independently move their joints, while PROM exercises are beneficial for patients with severe motor impairments who cannot perform active movements [44]. In our study, the values of upper limb AROM indicate that patients had sufficiently good motor control to perform the VR exercises. This suggests that the improvements observed are likely attributable not only to the effectiveness of the rehabilitation program but also to the patients’ ability to actively engage with the VR system. Taking into consideration the MMSE mean value of 27.50 (ranging from 25 to 30), this indicates that the patients did not have significant cognitive deficits and possessed the cognitive capacity to understand the exercises and instructions. The significant gains across various joints demonstrate that the patients could successfully participate in and benefit from the VR exercises, further reinforcing the role of VR as a viable tool for enhancing motor recovery in individuals with stroke.

One of the most common complications of stroke is shoulder pain, which often creates activity limitations, especially during dressing and ambulation [45]. In our study, shoulder pain, as measured by the Numerical Rating Scale (NRS), was significantly reduced after combining CnvT and VR therapy, from 2.28 (1.85) to 2.28 (1.85), with a *p*-value of ˂0.001. Some VR technologies that incorporate relaxation programs can effectively reduce pain during rehabilitation. Different virtual environments can have varying effects on pain perception [46]. VR can enhance patient engagement in rehabilitation programs by providing an immersive and distracting experience [47]. Lowering pain levels can help maintain motivation and adherence to rehabilitation programs. It has been proven that mental imagery improves central neuropathic pain in spinal cord injury and that stress and depression reduce the efficiency of this rehabilitation technique. VR can enhance mental imagery techniques by reducing depression and stress levels through relaxation programs [48,49]. Physical therapy, including transcutaneous electrical stimulation (TENS), helps reduce pain, and botulinum toxin (BoNT) injections can also effectively alleviate pain [50,51]. In our study, all patients with pain received TENS as part of CnVT, and 8 patients benefited from BoNT injections, with an average time since the last injection of 1.81 months.

WHODAS 2.0 is a reliable tool for measuring functioning, limitation of activity, and restriction of participation in stroke survivors across all six domains. It demonstrates strong internal consistency, with Cronbach’s alpha values ranging from 0.83 to 0.99. Additionally, the WHODAS 2.0 questionnaire correlates strongly with the Functional Independence Measure (FIM), a widely used tool for assessing disability in stroke patients [52]. In our study, the disability scores decreased across all six domains, with a significant reduction particularly observed in the Cognition (D1) domain (*p* = 0.011). The reduction in disability scores across all six domains—Mobility (D2), Self-Care (D3), Getting along with people (D4), Life activities (D5), and Participation in society (D6)—suggests that the combination of CnvT with VR enhances functionality in stroke survivors, having a positive impact on social interactions and interpersonal relationships. It also shows beneficial effects on daily living tasks and occupational roles, even though these changes are not statistically significant. Additionally, the improvement observed in the Cognition (D1) domain further indicates that VR training may effectively promote neuroplasticity, contributing to overall recovery [53,54].

A rehabilitation program that works best would include the following: (1) the patient actively participating in the training process; (2) training that challenges the person’s skills in addition to requiring many repetitions; (3) motivation and reward; (4) training and practice over an extended period of time; (5) careful planning of the training in relation to other activities; and (6) modification of other habits like sleep and diet [55]. The VR TRAVEE system incorporates some of these elements, such as active participation and challenging training with many repetitions, even without the therapist present. However, CnvT remains a foundational pillar of rehabilitation and long-term recovery for stroke survivors.

The limitations of the present study are as follows: firstly, the lack of a control group, which makes it challenging to attribute the observed improvements only to the VR intervention; secondly, the limited sample size, 14 patients, which may restrict how broadly the results may be applied. These two limitations raise questions about the applicability of the findings. Another limitation is the short (30-day) follow-up period, which lacks the sustainability of the improvements made with the VR system. However, considering that the mean time since stroke onset was 26.25 months (5.25 to 31.5), this may not be a critical limitation. The inclusion criteria, which excluded patients with severe cognitive impairment (required MMSE ≥ 25), may select a group of patients that is more likely to recover after rehabilitation therapy. Furthermore, the specificity of the TRAVEE VR system used, with games designed for different motor functions, may limit the generalizability of the results to other VR systems. Limited functional assessment tools were used in the study, specifically WHODAS 2.0. However, it is important to note that WHODAS 2.0 is a validated and comprehensive tool for assessing disability across multiple domains, which could be seen as a strength. At this moment, two patients have received this system for use at home. We have a feedback questionnaire in which most patients claim that it is an experience they would like to repeat and that they want to be part of the usual rehabilitation program. The feedback questionnaire will be published in a further study. Lastly, an important strength to mention is that as a pilot study, this research serves as an important preliminary investigation.

## 5. Conclusions

Using immersive VR and, respectively, the TRAVEE system in association with CnvT as a rehabilitation technique, stroke patients with residual hemiparesis can significantly improve upper limb motor function, as measured by active range of motion. Additionally, disability scores assessed by WHODAS 2.0 showed reductions across all six domains, with the Cognition domain being the only one to decrease significantly, suggesting the potential of VR in enhancing neuroplasticity. Further studies with larger sample sizes and extended follow-up periods are necessary to establish standardized protocols for VR use in stroke rehabilitation.

## Figures and Tables

**Table 1:** Presentation, screenshot, and description of all 10 game-based exercises used in this study, namely, Balls game, Fires game, Rocket game, Cooking game, Fruit to Basket game, Fruit to Mouth game, Road game, Piano Keys game, Piano Touch game, and Diorama game.

Game	Description	Gameplay Screenshot	Impairments/ Movements Targeted
Balls	The patients are asked to touch a set of balls coming toward them in the virtual environment.	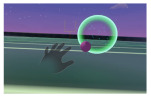	Extension of the shoulder Flexion and extension of the wrist
Fruits	The patients are asked to “pick” a set of fruits floating in the air and place them in a basket in front of them.	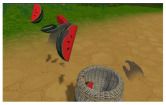	Extension and supination
	An alternative to the game mentioned above is one where the patients are asked to “pick” the fruits and bring them to their mouths.		Flexion and pronation
Road	The patients are asked to touch the entire perimeter of a curve with their hands. This curve is shaped like a branch and fills in with leaves, as shown in the adjacent figure.	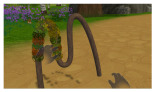	Supination and pronation
Rocket	The patients are asked to raise their hands to a certain height to launch a rocket aimed at a little monster. At regular intervals, a little monster appears in front of the patients and moves toward them. The patients have a few seconds to raise their hands to the required height to launch the rocket that stops the monster.	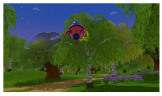	Abduction and flexion of the shoulder
Fires	The patients are asked to control a stream of water coming from their hands to extinguish flames at the windows of a building. There are several types of games.	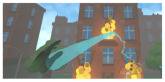	
Cooking	The patients are asked to prepare burgers on a tray in front of them. Ingredients for the burgers appear on the left and right sides of the tray, and the patients must move them to a specific position in the center of the tray to layer the burger. The first and last ingredients are always bun slices.	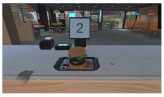	Internal and external rotation of the shoulder
Piano	The patients are asked to touch the keys of a keyboard to play the piano. Markers indicate which key to press coming toward the patients.	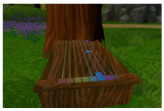	Flexion and extension of the wrist
	An alternative version of the game described above is where the patients are only asked to touch the markers coming toward them.		
Diorama	The patients are asked to place 14 pieces of diorama. The pieces appear on the left or right side of the patients and must be placed in a fixed position on a model.	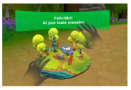	Flexion and extension of the wrist

**Table 2:** Characteristics of patients.

Characteristic	*n* = 14
Age (years) ± mean (SD)	55.29 ± 8.6
Females	14.27%
Males	85.71%
Urban	71.42%
Rural	28.57%
Education (years) ± mean (SD)	13.36 ± 2.72
Marital status (married)	70.4%
Employment status (retirees)	78.57%
Stroke	
Ischemic	78.57%
Hemorrhagic	21.42%
Left side affected	85.71%
Right side affected	14.27%
Months since the onset of stroke, median (IQR)	26.25 (5.25 to 31.5)
Smoker	50%
Alcohol consumer	21.42
MMSE, median (IQR)	27.50 (25 to 30)
Number of patients injected with Botulinum toxin	8
Average time since last injection (months)	1.81

SD—standard deviation, IQR—interquartile range.

**Table 3:** Patients’ comorbidities.

Characteristic	*n* = 14
HBP (%)	92.86%
Type 2 Diabetes Mellitus (%)	21.43%
Dyslipidemia	85.71%
Atrial Fibrillation	14.29%

HBP—high blood pressure.

**Table 4:** Modified Ashworth Scale assessed pre-treatment (T0) and post-treatment (T1).

Clinical Endpoints	T0 Median (IQR)	T1 Median (IQR)	*p*
MAS shoulders adductors	1.5 (1.0 to 2.0)	1.0 (1.0 to 1.5)	0.02
MAS elbow flexors	1.5 (1.0 to 2.0)	1.0 (1.0 to 1.5)	0.008
MAS pronators	1 (1 to 1.5)	1 (1 to 1.5)	0.083
MAS wrist flexors	1.5 (0.75 to 2.0)	1 (0.75 to 1.5)	0.023
MAS fingers flexors	1.25 (0.0 to 1.62)	1 (0.0 to 1.5)	0.034

IQR—interquartile range; MAS—Modified Ashworth Scale.

**Table 5:** Range of motion and shoulder pain assessed pre-treatment (T0) and post-treatment (T1).

Clinical Endpoints	T0 Media (SD)	T1 Media (SD)	*p*
AROM abduction (°)	98.57 (18.95)	102.28 (18.28)	˂0.001
AROM shoulder flexion (°)	101.07 (31.90)	104.42 (30.85)	˂0.001
AROM internal rotation (°)	53 (5.96)	54 (5.96)	˂0.001
AROM external rotation (°)	41.07 (28.07)	45.28 (26.28)	˂0.001
AROM elbow flexion (°)	135.28 (16.16)	136.14 (14.26)	˂0.001
AROM pronation (°)	80.07 (3.51)	80.28 (3.60)	˂0.001
AROM supination (°)	61.28 (28.26)	64.28 (26.01)	˂0.001
AROM wrist flexion (°)	65.28 (14.77)	65.71 (14.67)	˂0.001
AROM wrist extension (°)	32.92 (22.27)	35.21 (21.41)	˂0.001
AROM metacarpophalangeal flexion (°)	83.00 (9.68)	83.71 (9.44)	˂0.001
AROM metacarpophalangeal extension (°)	7.64 (8.68)	10.42 (8.13)	˂0.001
NRS shoulder	2.28 (1.85)	1.68 (1.27)	˂0.001

SD—standard deviation; AROM—active range of motion; NRS—Numeric Rating Scale.

**Table 6:** WHODAS 2.0 across all six domains.

Disability Categories		T0 Disability Percentage	30 Days Disability Percentage	*p*-Value
Cognition		23.21%	14.88%	0.011
Mobility		39.29%	37.50%	0.714
Self-care		41.07%	36.16%	0.366
Getting along with people		27.86%	21.07%	0.142
Life activities	Household	65.63%	0.363	0.363
	Work or school activities	54.69%	0.391	0.392
Participation in society		34.82%	31.55%	0.340
Overall disability		40.94%	36.30%	0.127

## Data Availability

The data supporting this study’s findings are available upon request from the authors. Please note that the data are also being utilized in an ongoing doctoral project evaluating novel rehabilitation assessment techniques.

## References

[1] Feigin V.L., Brainin M., Norrving B., Martins S., Sacco R.L., Hacke W., Fisher M., Pandian J., Lindsay P. (2022). World Stroke Organization (WSO): Global Stroke Fact Sheet 2022. Int. J. Stroke.

[2] GBD 2019 Stroke Collaborators (2021). Global, Regional, and National Burden of Stroke and Its Risk Factors, 1990–2019: A Systematic Analysis for the Global Burden of Disease Study 2019. Lancet Neurol..

[3] Feigin V.L., Norrving B., Mensah G.A. (2017). Global Burden of Stroke. Circ. Res..

[4] World Health Organization International Classification of Functioning, Disability, and Health (2007). Children & Youth Version: ICF-CY.

[5] Miller E.L., Murray L., Richards L., Zorowitz R.D., Bakas T., Clark P., Billinger S.A. (2010). Comprehensive Overview of Nursing and Interdisciplinary Rehabilitation Care of the Stroke Patient: A Scientific Statement from the American Heart Association. Stroke.

[6] Langhorne P., Coupar F., Pollock A. (2009). Motor Recovery after Stroke: A Systematic Review. Lancet Neurol..

[7] Warburton E., Alawneh J.A., Clatworthy P.L., Morris R.S. (2010). Stroke Management. BMJ Clin. Evid..

[8] Wade D.T. (1992). Measurement in Neurological Rehabilitation. Curr. Opin. Neurol..

[9] Dobkin B., Carmichael T. (2005). Principles of Recovery after Stroke. Recovery After Stroke.

[10] Levin M.F., Kleim J.A., Wolf S.L. (2009). What Do Motor “Recovery” and “Compensation” Mean in Patients Following Stroke?. Neurorehabilit. Neural Repair.

[11] Dancause N., Nudo R.J. (2011). Shaping Plasticity to Enhance Recovery after Injury. Prog. Brain Res..

[12] Kwakkel G., Kollen B., Lindeman E. (2004). Understanding the Pattern of Functional Recovery after Stroke: Facts and Theories. Restor. Neurol. Neurosci..

[13] Levin M.F., Weiss P.L., Keshner E.A. (2015). Emergence of Virtual Reality as a Tool for Upper Limb Rehabilitation: Incorporation of Motor Control and Motor Learning Principles. Phys. Ther..

[14] Viau A., Feldman A.G., McFadyen B.J., Levin M.F. (2004). Reaching in Reality and Virtual Reality: A Comparison of Movement Kinematics in Healthy Subjects and in Adults with Hemiparesis. J. Neuroeng. Rehabil..

[15] Thornton M., Marshall S., McComas J., Finestone H., McCormick A., Sveistrup H. (2005). Benefits of Activity and Virtual Reality Based Balance Exercise Programmes for Adults with Traumatic Brain Injury: Perceptions of Participants and Their Caregivers. Brain Inj..

[16] Ferche O., Moldoveanu A., Cinteza D., Toader C., Moldoveanu F., Voinea A., Taslitchi C. From Neuromotor Command to Feedback: A Survey of Techniques for Rehabilitation through Altered Perception. Proceedings of the 2015 E-Health and Bioengineering Conference.

[17] Chiaramonte R., D’Amico S., Caramma S., Grasso G., Pirrone S., Ronsisvalle M.G., Bonfiglio M. (2024). The Effectiveness of Goal-Oriented Dual Task Proprioceptive Training in Subacute Stroke: A Retrospective Observational Study. Ann. Rehabil. Med..

[18] Cameirao M.S., i Badia S.B., Duarte E., Frisoli A., Verschure P.F. (2012). The Combined Impact of Virtual Reality Neurorehabilitation and Its Interfaces on Upper Extremity Functional Recovery in Patients with Chronic Stroke. Stroke.

[19] Moldoveanu A., Ferche O.-M., Moldoveanu F., Lupu R.G., Cinteză D., Constantin Irimia D., Toader C. (2019). The TRAVEE System for a Multimodal Neuromotor Rehabilitation. IEEE Access.

[20] Gibson L.M., Brazzelli M., Thomas B.M., Sandercock P.A. (2010). A Systematic Review of Clinical Trials of Pharmacological Interventions for Acute Ischaemic Stroke (1955–2008) That Were Completed, but Not Published in Full. Trials.

[21] Zampolini M., Selb M., Boldrini P., Branco C.A., Golyk V., Hu X., Kiekens C., Negrini S., Nulle A., Oral A. (2022). The Individual Rehabilitation Project as the Core of Person-Centered Rehabilitation: The Physical and Rehabilitation Medicine Section and Board of the European Union of Medical Specialists Framework for Rehabilitation in Europe. Eur. J. Phys. Rehabil. Med..

[22] Ustun T.B., Kostanjesek N., Chatterji S., Rehm J., Üstün T.B., Kostanjsek N., Chatterji S., Rehm J. (2010). Measuring Health and Disability: Manual for WHO Disability Assessment Schedule (WHODAS 2.0).

[23] Potcovaru C.-G., Salmen T., Bîgu D., Săndulescu M.I., Filip P.V., Diaconu L.S., Pop C., Ciobanu I., Cinteză D., Berteanu M. (2024). Assessing the Effectiveness of Rehabilitation Interventions through the World Health Organization Disability Assessment Schedule 2.0 on Disability—A Systematic Review. J. Clin. Med..

[24] Unit Calculator. https://alcoholchange.org.uk/alcohol-facts/interactive-tools/unit-calculator.

[25] White I.R., Altmann D.R., Nanchahal K. (2002). Alcohol Consumption and Mortality: Modelling Risks for Men and Women at Different Ages. BMJ.

[26] Caraiman S., Stan A., Botezatu N., Herghelegiu P., Lupu R.G., Moldoveanu A. Architectural Design of a Real-Time Augmented Feedback System for Neuromotor Rehabilitation. Proceedings of the 2015 20th International Conference on Control Systems and Computer Science.

[27] Harb A., Kishner S. (2023). Modified Ashworth Scale. StatPearls [Internet].

[28] Norkin C.C., White D.J. (2016). Measurement of Joint Motion: A Guide to Goniometry.

[29] WHO Disability Assessment Schedule (WHODAS 2.0). https://www.who.int/standards/classifications/international-classification-of-functioning-disability-and-health/who-disability-assessment-schedule.

[30] Faria A.L., Cameirão M.S., Couras J.F., Aguiar J.R.O., Costa G.M., Bermúdez i Badia S. (2018). Combined Cognitive-Motor Rehabilitation in Virtual Reality Improves Motor Outcomes in Chronic Stroke—A Pilot Study. Front. Psychol..

[31] Henderson A., Korner-Bitensky N., Levin M. (2007). Virtual Reality in Stroke Rehabilitation: A Systematic Review of Its Effectiveness for Upper Limb Motor Recovery. Top. Stroke Rehabil..

[32] Dąbrowská M., Pastucha D., Janura M., Tomášková H., Honzíková L., Baníková Š., Filip M., Fiedorová I. (2023). Effect of Virtual Reality Therapy on Quality of Life and Self-Sufficiency in Post-Stroke Patients. Medicina.

[33] Kuerbis A., Mulliken A., Muench F., Moore A., Gardner D. (2017). Older Adults and Mobile Technology: Factors That Enhance and Inhibit Utilization in the Context of Behavioral Health. Publ. Res..

[34] Hodgson J.C., Benattayallah A., Hodgson T.L. (2014). The Role of the Dominant versus the Non-Dominant Hemisphere: An fMRI Study of Aphasia Recovery Following Stroke. Aphasiology.

[35] Salmen T., Serbanoiu L.-I., Bica I.-C., Serafinceanu C., Muzurović E., Janez A., Busnatu S., Banach M., Rizvi A.A., Rizzo M. (2023). A Critical View over the Newest Antidiabetic Molecules in Light of Efficacy—A Systematic Review and Meta-Analysis. Int. J. Mol. Sci..

[36] Oprea E., Berteanu M., Cintezã D., Manolescu B.N. (2013). The Effect of the ALAnerv Nutritional Supplement on Some Oxidative Stress Markers in Postacute Stroke Patients Undergoing Rehabilitation. Appl. Physiol. Nutr. Metab..

[37] Weintraub M.S., Rosen Y., Otto R., Eisenberg S., Breslow J.L. (1989). Physical Exercise Conditioning in the Absence of Weight Loss Reduces Fasting and Postprandial Triglyceride-Rich Lipoprotein Levels. Circulation.

[38] Carraro E., Schilirò T., Biorci F., Romanazzi V., Degan R., Buonocore D., Verri M., Dossena M., Bonetta S., Gilli G. (2018). Physical Activity, Lifestyle Factors and Oxidative Stress in Middle Age Healthy Subjects. Int. J. Environ. Res. Public Health.

[39] Doussoulin A., Rivas C., Bacco J., Sepúlveda P., Carvallo G., Gajardo C., Soto A., Rivas R. (2020). Prevalence of Spasticity and Postural Patterns in the Upper Extremity Post Stroke. J. Stroke Cerebrovasc. Dis..

[40] Seok Ryu J., Woo Lee J., Il Lee S., Ho Chun M. (2010). Factors Predictive of Spasticity and Their Effects on Motor Recovery and Functional Outcomes in Stroke Patients. Top. Stroke Rehabil..

[41] Lundström E., Terént A., Borg J. (2008). Prevalence of Disabling Spasticity 1 Year after First-ever Stroke. Eur. J. Neurol..

[42] Chheang V., Lokesh R., Chaudhari A., Wang Q., Baron L., Kiafar B., Doshi S., Thostenson E., Cashaback J., Barmaki R.L. (2023). Immersive Virtual Reality and Robotics for Upper Extremity Rehabilitation. arXiv.

[43] Hosseini Z.-S., Peyrovi H., Gohari M. (2019). The Effect of Early Passive Range of Motion Exercise on Motor Function of People with Stroke: A Randomized Controlled Trial. J. Caring Sci..

[44] Indrawati I., Sudiana K., Sajidin M. Active Passive and Active-Assistive Range of Motion (ROM) Exercise to Improve Muscle Strength in Post Stroke Clients: A Systematic Review. Proceedings of the 9th International Nursing Conference: Nurses at The Forefront Transforming Care, Science and Research.

[45] Lindgren I., Jönsson A.-C., Norrving B., Lindgren A. (2007). Shoulder Pain After Stroke: A Prospective Population-Based Study. Stroke.

[46] Funao H., Tsujikawa M., Momosaki R., Shimaoka M. (2021). Virtual Reality Applied to Home-Visit Rehabilitation for Hemiplegic Shoulder Pain in a Stroke Patient: A Case Report. J. Rural Med..

[47] Shahrbanian S., Simmonds M.J. (2008). Effects of Different Virtual Reality Environments on Experimental Pain Rating in Post-Stroke Individuals with and without Pain in Comparison to Pain Free Healthy Individuals. Annu. Rev. Cybertherapy Telemed..

[48] Kaur J., Ghosh S., Sahani A.K., Sinha J.K. (2020). Mental Imagery as a Rehabilitative Therapy for Neuropathic Pain in People with Spinal Cord Injury: A Randomized Controlled Trial. Neurorehabilit. Neural Repair.

[49] Kaur J., Ghosh S., Singh P., Dwivedi A.K., Sahani A.K., Sinha J.K. (2022). Cervical Spinal Lesion, Completeness of Injury, Stress, and Depression Reduce the Efficiency of Mental Imagery in People With Spinal Cord Injury. Am. J. Phys. Med. Rehabil..

[50] Poenaru D., Sandulescu M.I., Cinteza D. (2023). Pain Modulation in Chronic Musculoskeletal Disorders: Botulinum Toxin, a Descriptive Analysis. Biomedicines.

[51] Trofin D., Salmen B.-M., Salmen T., Trofin D.M., Reurean-Pintilei D. (2024). Advancing the Diagnosis of Diabetic Neuropathies: Electrodiagnostic and Skin Autofluorescence Methods. J. Pers. Med..

[52] Küçükdeveci A.A., Kutlay Ş., Yıldızlar D., Öztuna D., Elhan A.H., Tennant A. (2013). The Reliability and Validity of the World Health Organization Disability Assessment Schedule (WHODAS-II) in Stroke. Disabil. Rehabil..

[53] Cheung K.L., Tunik E., Adamovich S.V., Boyd L.A. (2014). Neuroplasticity and Virtual Reality. Virtual Real. Phys. Mot. Rehabil..

[54] Hao J., Xie H., Harp K., Chen Z., Siu K.-C. (2022). Effects of Virtual Reality Intervention on Neural Plasticity in Stroke Rehabilitation: A Systematic Review. Arch. Phys. Med. Rehabil..

[55] Nielsen J.B., Willerslev-Olsen M., Christiansen L., Lundbye-Jensen J., Lorentzen J. (2015). Science-Based Neurorehabilitation: Recommendations for Neurorehabilitation from Basic Science. J. Mot. Behav..

